# Nitazoxanide, an antiprotozoal drug, inhibits late-stage autophagy and promotes ING1-induced cell cycle arrest in glioblastoma

**DOI:** 10.1038/s41419-018-1058-z

**Published:** 2018-10-09

**Authors:** Xiaoxiong Wang, Chen Shen, Zhendong Liu, Fei Peng, Xin Chen, Guang Yang, Daming Zhang, Zhiqin Yin, Jichao Ma, Zhixing Zheng, Boxian Zhao, Huailei Liu, Ligang Wang, Jianing Wu, Dayong Han, Kaikai Wang, Chen Zhong, Xu Hou, Wenyang Zhao, Mengting Shu, Xinzhuang Wang, Shiguang Zhao

**Affiliations:** 1 Department of Neurosurgery, The First Affiliated Hospital of Harbin Medical University, No. 23 Youzheng Street, Nangang District, 150001 Harbin, Heilongjiang Province People’s Republic of China; 2 Institute of Brain Science, Harbin Medical University, No. 23 Youzheng Street, Nangang District, 150001 Harbin, Heilongjiang Province People’s Republic of China; 3 Institute of Neuroscience, Sino-Russian Medical Research Center, Harbin Medical University, No. 23 Youzheng Street, Nangang District, 150001 Harbin, Heilongjiang Province People’s Republic of China; 4 Department of Pharmacology, The State-Province Key Laboratories of Biomedicine-Pharmaceutics of China, College of Pharmacy of Harbin Medical University, No. 157 Baojian Street, Nangang District, 150001 Harbin, Heilongjiang Province People’s Republic of China

## Abstract

Glioblastoma is the most common and aggressive primary brain tumor in adults. New drug design and development is still a major challenge for glioma treatment. Increasing evidence has shown that nitazoxanide, an antiprotozoal drug, has a novel antitumor role in various tumors and exhibits multiple molecular functions, especially autophagic regulation. However, whether nitazoxanide-associated autophagy has an antineoplastic effect in glioma remains unclear. Here, we aimed to explore the underlying molecular mechanism of nitazoxanide in glioblastoma. Our results showed that nitazoxanide suppressed cell growth and induced cell cycle arrest in glioblastoma by upregulating ING1 expression with a favorable toxicity profile. Nitazoxanide inhibited autophagy through blockage of late-stage lysosome acidification, resulting in decreased cleavage of ING1. A combination with chloroquine or Torin1 enhanced or impaired the chemotherapeutic effect of nitazoxanide in glioblastoma cells. Taken together, these findings indicate that nitazoxanide as an autophagy inhibitor induces cell cycle arrest in glioblastoma via upregulated ING1 due to increased transcription and decreased post-translational degradation by late-stage autophagic inhibition.

## Introduction

Glioma is the most common type of malignant brain tumor in adults, accounting for 27% of all primary central nervous system (CNS) tumors. Among these, glioblastoma multiforme (GBM, WHO grade IV) is the most lethal CNS tumor and is characterized by excessive proliferation, aggressive invasion and high resistance to conventional therapies^1,2^. Chemotherapy is widely used in adjuvant approaches for the treatment of brain tumors, especially glioma. Currently, numerous antineoplastic drugs, such as temozolomide, carmustine wafer and bevacizumab, have been approved for treatment of glioma; these drugs alter MGMT promoter methylation, DNA and RNA crosslinking, cell cycle arrest, VEGF, and autophagy^2,3^. Despite these current advances in the clinical treatment of glioma, little improvement has been made in the median survival time of initially diagnosed GBM patients, which is 15–18 months on average^2^. Therefore, identification and development of new therapeutics for glioma patients is urgently needed.

Drug repurposing, also known as drug repositioning is a novel therapeutic switching strategy that has gained popularity in the development of new agents^4,5^. The repurposing of existing treatments, such as sildenafil and metformin, for alternative disorders can save time and money in drug design and development^6^. Nitazoxanide (NTZ), an antiprotozoal drug used against protozoan, bacterial or viral infections such as Cryptosporidia, Helicobacter or Hepatitis C, has shown a wide spectrum of pharmacological functions in infectious and neoplastic diseases^7–9^. However, the chemotherapeutic role of NTZ in glioma remains unclear.

To date, the pharmacological effects of NTZ include mediating the unfolded protein response (UPR), reversing chemotherapy detoxification, targeting the c-Myc signaling pathway, stimulating the immune response, and especially regulating autophagy^9–13^. Autophagy is an intracellular lysosomal degradation process regulated by a variety of highly conserved autophagy-related genes (ATGs) through different mechanisms^14^. This homeostatic process could affect or be induced by multiple cellular stressors and signaling pathways involved in nutrient and growth factor status, energy sensing, hypoxia, oxidative and endoplasmic reticulum (ER) stress, pathogen infection, or chemotherapy resistance^15,16^. Interestingly, inhibition or activation of autophagy may produce synergistic or contradictory effects on cancer therapy depending on the cellular context^17,18^. Thus, whether autophagy is involved in the chemotherapeutic effects of NTZ and whether NTZ combined with inhibition or activation of autophagy enhances or impairs the chemotherapeutic efficacy still need to be confirmed.

In the present study, we demonstrated the therapeutic efficacy of NTZ either alone or combined with an autophagy inducer or inhibitor on glioma growth in vitro and in vivo. We further screened target genes of NTZ and investigated the underlying molecular mechanism of NTZ-associated autophagic suppression in glioma treatment.

## Results

### NTZ decreases glioma cell viability and proliferation

**Fig. 1:**
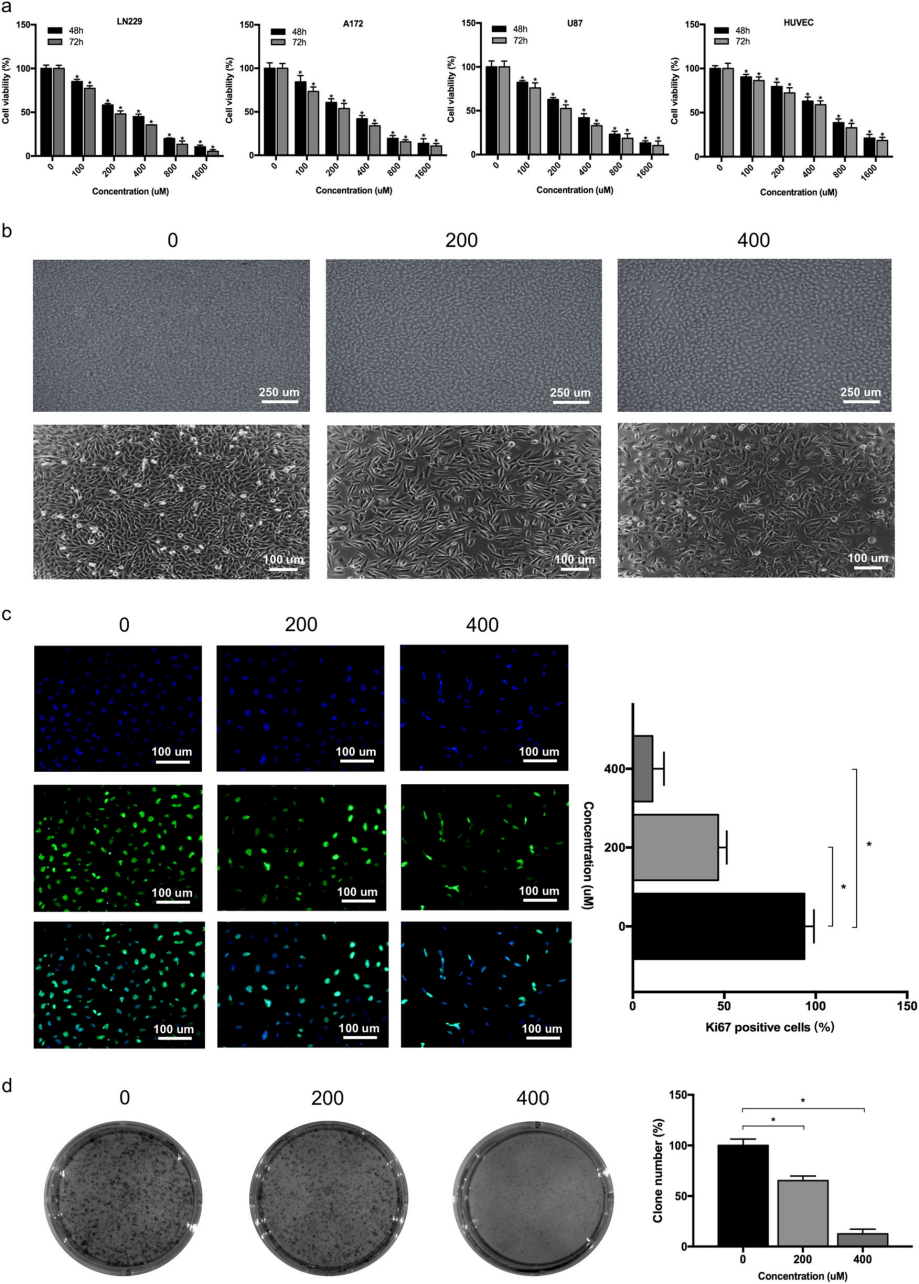
NTZ inhibits glioma cell growth in vitro. **a** Cell viability of LN229, A172, U87, and HUVECs determined by MTT assays after 48 h and 72 h of NTZ treatment. **b** Phase contrast microscopy of LN229 cells inhibited by NTZ. Scale bar represents 100 or 250 μm. **c** Fluorescence microscopy of Ki67 expression after treatment of the LN229 cell line with NTZ at concentrations of 0, 200, and 400 μM for 48 h. Scale bar represents 100 μM. **d** Colony formation assays of LN229 cells treated with 0, 200, and 400 μM NTZ. The experiments were repeated 3 times independently, and the bars represent SD. The data were normalized with control group (*P < 0.05)

To investigate the effect of NTZ on glioma cell viability, we exposed LN229, U87, A172, and HUVECs to different NTZ concentrations ranging from 100 to 1600 µM for 48 h and 72 h. As shown in Fig. 1a, NTZ inhibited cell proliferation in the 4 cell lines in a dose-dependent and time-dependent manner, which significantly reduced cell viability in the 48 h and 72 h groups. The 48 h IC_50_ values of NTZ were 383.39 μM for LN229, 398.66 μM for A172, 411.72 μM for U87 and 659.93 μM for HUVECs. Inhibition of cell proliferation was augmented after 48 h of NTZ treatment as shown by light microscopy (Fig. 1b). The fluorescence results further indicated that expression of the proliferative marker Ki67 was decreased in the LN229 cell line (Fig. 1c). Similarly, colony formation assays showed that colony formation was significantly decreased after NTZ exposure (Fig. 1d). These results indicate that NTZ exhibits cytotoxicity and inhibits cell growth in glioma cells.

### Identification of ING1 as a therapeutic target of NTZ and a predictor of survival in GBM patients

**Fig. 2:**
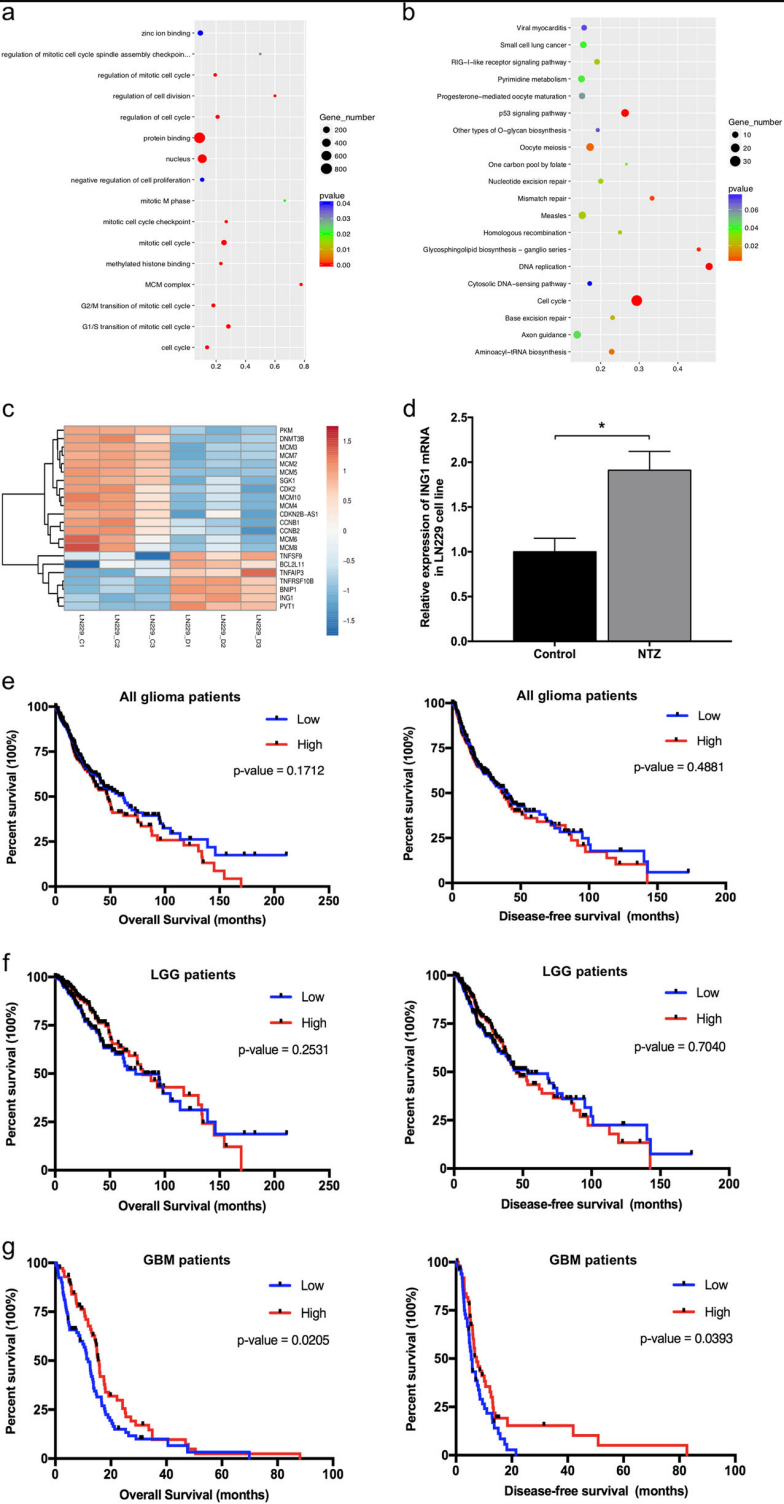
Identification of therapeutic targets after NTZ treatment and survival analysis of ING1 in glioma patients. **a–b** GO and KEGG analysis of significant target genes. **c** Heatmap of altered genes after NTZ treatment. **d** The relative expression of ING1 in LN229 cells after 48 h NTZ treatment. The experiments were repeated 3 times independently, and the bars represent SD. The data were normalized with control group (*P < 0.05). **e** Kaplan–Meier survival analysis of ING1 for all glioma patients (662 glioma tissue samples). **f** Kaplan–Meier survival analysis of ING1 for LGG glioma patients (510 LGG tissue samples). Low-grade glioma LGG. **g** Kaplan–Meier survival analysis of ING1 for GBM patients (152 GBM tissue samples)

To identify potential genes and pathways regulated by NTZ, we performed a RNA-seq analysis to investigate the gene expression profile and differential pathways after NTZ treatment (Fig. 2 and Table S1-4). There were 5523 differentially expressed transcripts (1981 upregulated transcripts and 3542 downregulated transcripts) and 1420 differentially expressed genes (722 upregulated genes and 698 downregulated genes), which are shown in Table S1-2. Gene Ontology (GO) analysis showed that G1/S and G2/M transition of mitotic cell cycles was significantly enriched in 405 GO functional terms (Fig. 2a). In addition, Kyoto Encyclopedia of Genes and Genomes (KEGG) enrichment pathway analysis revealed that the cell cycle pathway was a major cell pathway influenced by NTZ among 15 differentially expressed pathways (Fig. 2b). Among the differentially expressed genes, ING1 was significantly overexpressed in the NTZ group (Fig. 2c). Similar to the RNA-seq results, the relative expression of ING1is higher in NTZ group than that in control group by the PCR analysis (Fig. 2d). Previous studies have shown ING1, a tumor suppressor, has a negative regulatory role on the cell cycle in various types of cancers^19^. Thus, we hypothesized that NTZ predominantly exerts its pharmacological function in glioma cell cycle arrest by upregulating ING1.

To assess the clinical correlation between ING1 expression and prognosis of glioma patients, we performed survival analysis of glioma RNA-seq data separated into high and low ING1 level groups according to the mean expression in TCGA database. Although there was no significant association between ING1 expression and survival in all glioma patients (Fig. 2e), low ING1 expression was identified as a poor prognostic factor of overall survival and disease-free survival in GBM patients but not low-grade glioma patients (Fig. 2f, g). The survival analysis suggests that ING1 serves as a tumor suppressor with a prognostic role in GBM patients.

### NTZ induces cell cycle arrest by upregulation of ING1

**Fig. 3:**
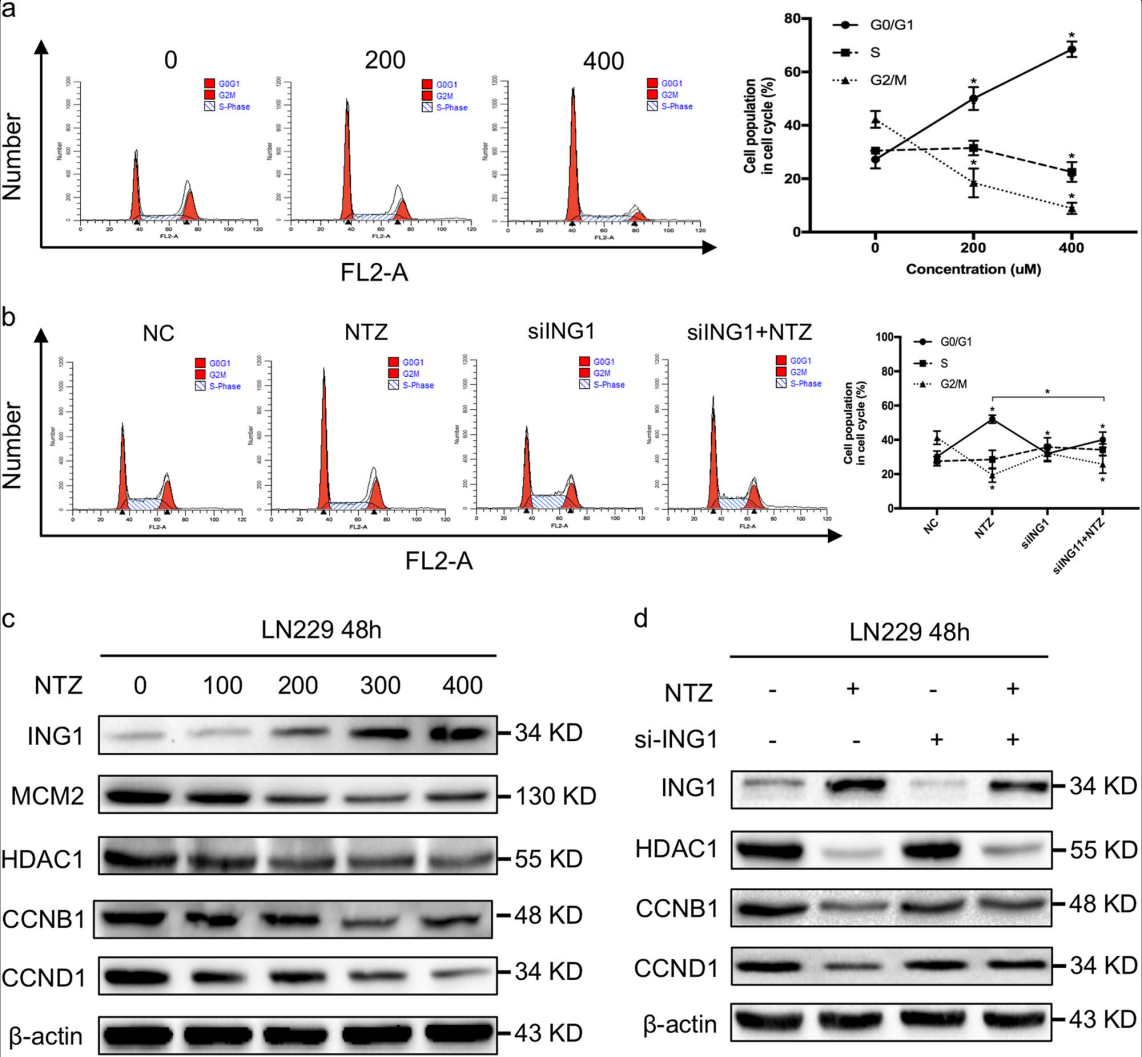
NTZ suppresses the cell cycle by upregulating ING1 expression. **a** The cell cycle distribution after treatment with different concentrations of NTZ for 48 h. **b** The cell cycle was assessed by flow cytometry after knockdown of ING1. **c** Dose-dependent effect of NTZ on cell cycle-related proteins. **d** The expression of cell cycle-related proteins in ING1-knockdown cells. The experiments were repeated 3 times independently, and the bars represent SD (*P < 0.05)

After the bioinformatics and survival analysis, we investigated whether increased ING1 induced cell cycle arrest in vitro. Then, the cell cycle distribution was assessed after 48 h of treatment with 0, 200, and 400 μM NTZ. As shown in Fig. 3a, the percentage of cells in G0/G1 phase increased from 27.19% to 68.53%. To further elucidate the underlying molecular mechanism, we examined the expression profile of cell cycle-related proteins. The results showed a dose-dependent decrease in cyclin B1, cyclin D1, MCM2, and HDAC1 expression but an increase in ING1 expression, similar to the high-throughput sequencing data above (Fig. 3c). Knockdown of ING1 rescued the G0/G1 phase arrest by upregulation of cyclin B1 and cyclin D1 expression (Fig. 3d). The percentage of cells in G0/G1 phase decreased from 52.06% to 40.05% after ING1 knockdown (Fig. 3b).

The histone deacetylase (HDAC) family, which includes HDAC1-11, maintains epigenetic modification of human histones^20^. Downregulation of HDAC1 induces cell cycle arrest and apoptosis in different tumors^21^. The combination of NTZ and si-ING1 increased HDAC1 expression compared with the NTZ group, which indicates that NTZ-induced ING1 overexpression may be involved in the regulation of HDAC1 under the context of NTZ (Fig. 3d). All these results show that NTZ induces G0/G1 phase arrest by upregulating ING1 in glioma.

### NTZ increases ING1 by blockage of late-stage autophagic flux

**Fig. 4:**
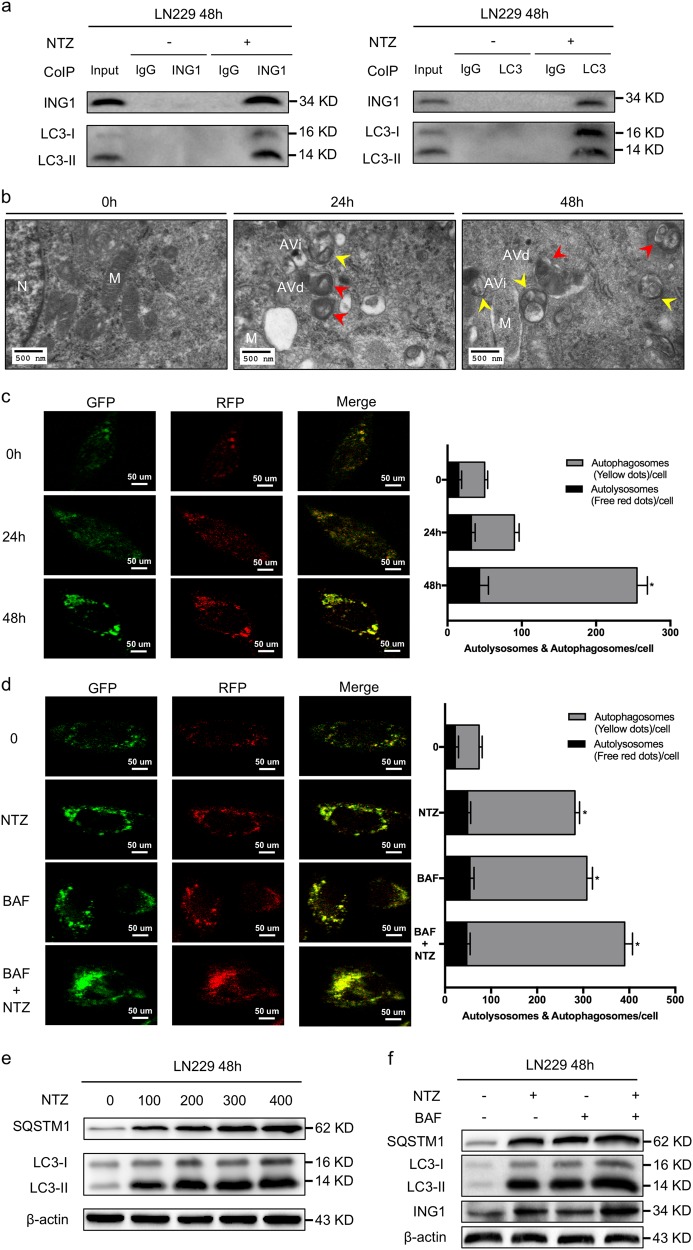
NTZ increases ING1 by blockage of late-stage autophagic flux. **a** Co-immunoprecipitation showing ING1 and LC3. LN229 cells were cultured with or without 200 μM NTZ for 48 h and immunoprecipitated with ING1 or LC3 antibody. **b** TEM photomicrographs of LN229 cells treated with 0 and 200 μM NTZ for 0 h, 24 h, and 48 h. N nucleus, M mitochondrion, AVs autophagic vacuoles, AVi initial AVs (yellow arrows), AVd late or degradative AVs (red arrows). Scale bar represents 500 nm. **c** The mRFP-GFP-LC3 distribution in LN229 cells cultured with 200 μM NTZ after 0 h, 24 h and 48 h was analyzed by confocal microscopy. Scale bar represents 50 μM. **d** The distribution of LN229 mRFP-GFP-LC3 immunofluorescence in cells cultured with 200 μM NTZ and 200 nM BAF after 48 h. Bafilomycin A1, BAF. Scale bar represents 50 μM. **e** Dose-dependent effect of NTZ on autophagy-related proteins. **f** Western blot showing SQSTM1 and LC3 levels in LN229 cells after treatment with 200 μM NTZ and 200 nM BAF for 48 h. The experiments were repeated 3 times independently, and the bars represent SD (*P < 0.05)

Increased transcriptional activity and decreased degradation may both enhance ING1 expression in glioma. Autophagy is a lysosome-dependent degradation and cell survival process in cellular protein degradation of tumors^22^. Therefore, we investigated whether autophagy was involved in ING1 degradation. Co-IP results showed that an interaction existed between ING1 and LC3, which suggested that the increased ING1 protein level was affected by NTZ-associated autophagy (Fig. 4a).

A previous study of *Mycobacterium tuberculosis* infection suggested that NTZ activated autophagy by inhibition of the mTORC1 signaling pathway^11^. To determine whether autophagic flux was also activated by NTZ in glioma, we observed autophagic vacuoles with their characteristic double membranes by TEM at 24 h and 48 h in the NTZ group (Fig. 4b). A block in lysosome trafficking also results in autophagic vacuole accumulation, and in this situation, an increase in autophagosomes may reflect a reduction in degradation. Thus, we must carefully determine that autophagosomes accumulate because of inhibition of autophagy instead of stimulation due to increased autophagic activity^14^. GFP-RFP-LC3 fluorescence assays are designed to monitor autophagic flux. The GFP signal is sensitive to the acidic conditions in lysosomes, in which RFP can stably exist. Therefore, colocalization of GFP and RFP fluorescence indicates a compartment without lysosome fusion, which usually suggests autophagosomes (yellow dots). In contrast, the RFP signal alone represents autolysosomes (red dots)^14,23^. GFP-RFP-LC3 double fluorescence showed that more yellow dots (autophagosomes) were aggregated than red dots (autolysosomes) after 48 h exposure to NTZ (Fig. 4c). SQSTM1 binds to LC3 and is selectively degraded in autolysosomes and is thus widely used as a marker for autophagic degradation. Blockage of autophagy is associated with increased levels of SQSTM1^14^. Similarly, we found that NTZ significantly inhibited autophagy with increased levels of LC3-II and SQSTM1 in a dose-dependent manner (Fig. 4e). Bafilomycin A1 is an inhibitor of vacuolar H^+^-ATPase isolated from *Streptomyces* species that blocks autophagy by inhibition of lysosomal acidification^24^. Increased yellow spots were observed in NTZ-treated cells, similar to the results in Bafilomycin A1-treated cells (Fig. 4d). In addition, increased numbers of yellow spots appeared in the merged section of NTZ and Bafilomycin A1-treated cells (Fig. 4d). SQSTM1 and ING1 levels were both increased in the NTZ and Bafilomycin A1 groups (Fig. 4f). These results indicate that NTZ upregulates ING1 by blocking the maturation of autolysosomes in autophagy.

### Regulation of autophagy affects the cytotoxic effect of NTZ

**Fig. 5:**
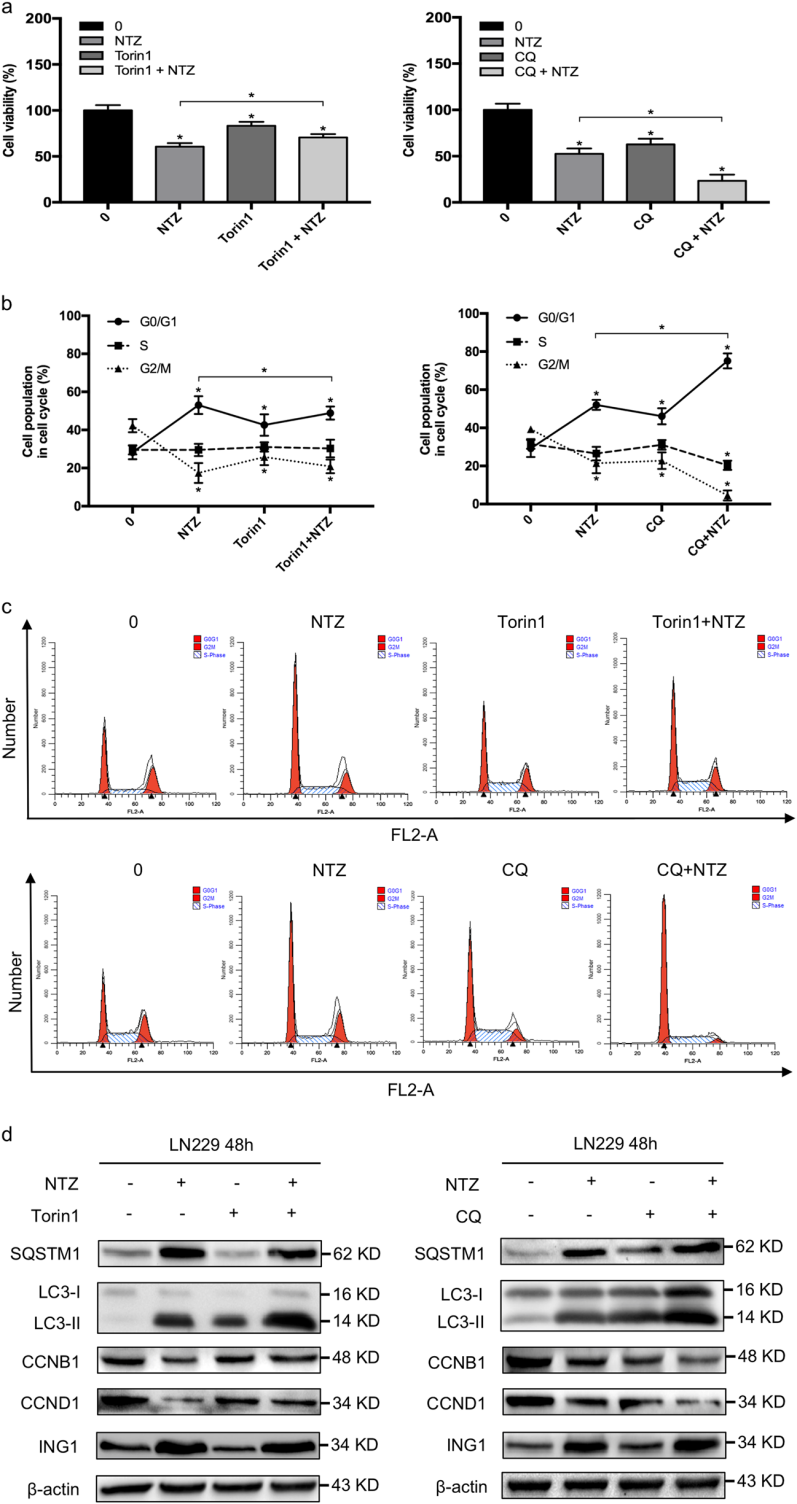
The cytotoxic effect of NTZ is affected by Torin1 and CQ. **a** LN229 cells were treated with 200 μM NTZ and/or 500 nM Trion1 or 30 mM CQ for 48 h and evaluated by MTT assays. Chloroquine, CQ. **b** The cell cycle distribution of LN229 cells treated as described above was assessed by flow cytometry. **c** Quantification of LN229 cell cycle distribution cultured with 200 μM NTZ and/or 500 nM Trion1 or 30 mM CQ for 48 h. **d** Western blot showing the cell cycle and autophagy-related proteins after 48 h of treatment with 200 μM NTZ and/or 500 nM Trion1 or 30 mM CQ. The experiments were repeated 3 times independently, and the bars represent SD. The data were normalized with control or matched group (*P < 0.05)

Inducers and inhibitors of autophagy, two types of contradictory drugs, sometimes exhibit synergistic or opposite effects on tumor chemotherapy when used in combination^25,26^. To investigate the potential role of autophagic inducers and inhibitors on NTZ cytotoxicity, we first used Torin1, a potent inhibitor of mTOR, combined with NTZ. MTT assays showed that cell viability increased after the Torin1 and NTZ combination compared to NTZ alone (Fig. 5a). Furthermore, cell cycle analysis also showed that Torin1 rescued G0/G1 phase arrest in the combined group compared to the NTZ group (45.89% vs. 54.06%) as shown in Fig. 5b, c. As shown in Fig. 5d, cyclin B1 and cyclin D1 expression decreased in NTZ-treated cells, whereas expression was clearly increased in cells treated with Torin1 and NTZ. Correspondingly, the ING1 level increased following the combination treatment compared with NTZ treatment alone (Fig. 5d). These data demonstrate that an autophagy inducer alleviates NTZ cytotoxicity in glioma.

To further evaluate whether the cytotoxicity of NTZ can be enhanced by autophagy inhibitors, we used chloroquine to block late-stage autophagic flux. After treatment, we found that the GBM cells were more sensitive to the chloroquine and NTZ combination than NTZ alone (Fig. 5a). In addition, the results of flow cytometry indicated that the combined treatment aggravated G0/G1 phase arrest compared to NTZ treatment (76.12% vs. 52.06%) as shown in Fig. 5b, c. Similar to the results of cell cycle analysis, the cyclin B1 and cyclin D1 levels decreased in the combined group compared with the NTZ group (Fig. 5d). Notably, chloroquine and NTZ significantly prevented ING1 degradation by interfering with lysosome acidification (Fig. 5d). Together, these results suggest that impaired clearance of ING1 by an autophagy inhibitor sensitizes GBM cells to NTZ treatment.

### NTZ inhibits glioma growth in vivo

**Fig. 6:**
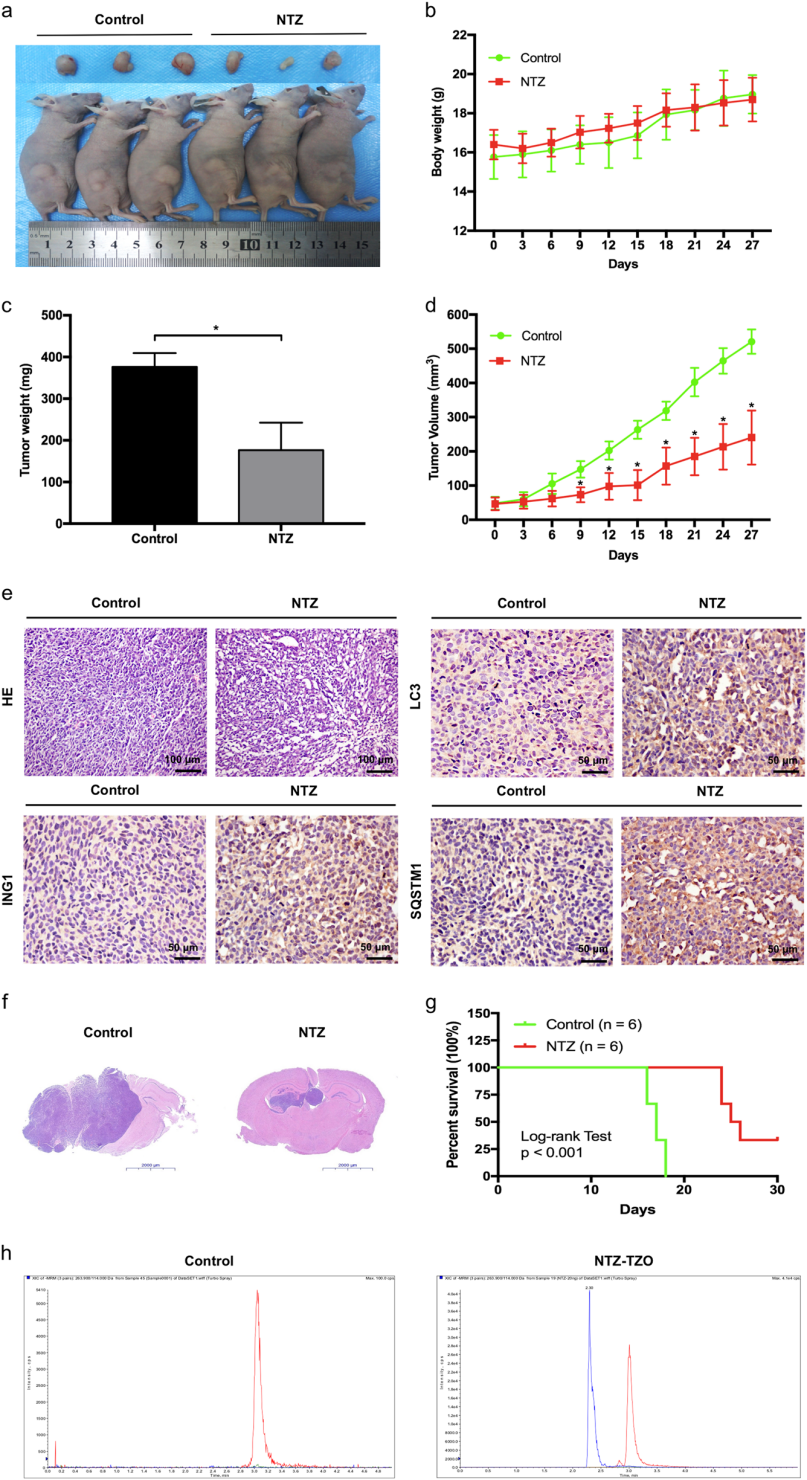
NTZ inhibits glioma growth in vivo. **a** Dissected tumors from a xenograft model with or without 27-day NTZ treatment after implantation. **b** Body weight changes of mouse models. **c** Tumor weight of two groups after the 27-day treatment. **d** Tumor volume changes of the two groups treated as described above. **e** HE and immunohistochemistry of ING, LC3, and SQSTM1 in vivo. Scale bar represents 50 or 100 μM. **f** The intracranial tumor size in orthotopic xenograft model. Scale bar represents 2000 μM. **g** The survival time of nude mice bearing glioma. **h** Representative LC–MS/MS chromatograms from TZO analysis of the brain homogenate samples. The statistic bars represent SD (*P < 0.05)

Next, we sought to assess the efficacy of NTZ therapy in vivo. As shown in Fig. 6a, inhibition of tumor growth after NTZ treatment was observed by gross inspection. In addition, body weight loss showed no significant difference between the two groups over 4 weeks (Fig. 6b). No common side effects of NTZ, such as vomiting, diarrhea and skin rash, were observed. Consistent with the gross observations, the therapeutic effect was further evaluated by tumor weight and volume. NTZ significantly inhibited tumor growth, which resulted in decreased tumor weight and volume in the NTZ group (Fig. 6c, d) In addition, elevated levels of ING1, LC3 and SQSTM1 were observed in the NTZ group compared with the control group (Fig. 6e). NTZ treatment by the orthotopic injection significantly inhibited intracranial tumor growth (Fig. 6f). More importantly, Kaplan–Meier curves analysis showed the median survival time was 25.5 days in NTZ group, whereas it was 17 days in control group, which indicated that NTZ significantly prolonged the survival time of nude mice bearing glioma (Fig. 6g). To investigate the efficiency and blood brain barrier (BBB) permeability of NTZ, we determined the concentration of the active metabolite of NTZ–tizoxanide (TZO) in glioma, we found NTZ can permeate BBB as shown in Fig. 6h and Table S5. All above, these data support that NTZ exhibits anti-glioma properties in vivo.

## Discussion

Cancer remains one of most common, complex, and deadly human diseases. The growing cost of global cancer care and treatment has resulted in a heavy financial burden in drug development, which has incentivized scientists and clinicians to repurpose or reposition “old drugs”^6,27^. Starting with an “old drug” provides a more efficient therapeutic switching way and fruitful basis for the discovery of new drugs, particularly glioma drugs^4,5,27^. For glioma treatment, a new use for an “old drug” has many benefits without the limitation of unknown safety and toxicity profiles. In this study, we demonstrated that ING1 was a favorable prognostic factor for GBM patients and that NTZ, which was previously used as an antiparasitic drug, could significantly inhibit glioma cells by upregulating transcription of the target gene, ING1, and decreasing autophagic degradation of ING1. Moreover, a combination of NTZ and chloroquine synergistically enhanced the cytotoxic effects on glioblastoma, suggesting NTZ may have potential chemotherapeutic value for glioblastoma.

NTZ, formally known as 2-(acetyloxy)-N-(5-nitro-2-thiazolyl) benzamide, was first synthesized and developed as an oral antiparasitic drug in the early 1970s^8^. Currently, NTZ has been widely recognized as a broad-spectrum anti-infection compound used against protozoa, bacteria and viruses^8,9^. With a notable pharmacokinetic safety profile, NTZ induces cytotoxic effects by modulating critical metabolic processes and pro-death signaling, especially the UPR, reactive oxygen species, chemotherapeutic detoxification, autophagy, and immune and inflammation responses in infectious diseases^8–10^. Recent evidence has shown that NTZ had an unexpected role in inhibiting tumor cell growth by suppression of c-Myc and induction of apoptosis, which suggests a new application in antitumor therapy^12,13^. However, whether NTZ has the same pharmacological profile in glioma is still unknown. In this study, we demonstrated that NTZ significantly suppressed glioma cells and showed a wide therapeutic window in HUVECs at a high inhibitory concentration, which indicates that NTZ exhibits selective inhibition toward rapid proliferation of glioma. Obstacles to the chemotherapeutic efficacy in CNS tumors include low drug penetration due to BBB, little free and available drug due to high plasma protein binding, and intrinsic and acquired chemoresistance^28^. One strategy of drug delivery to brain tumors is high-dose chemotherapy, but this can have adverse effects—dose-limiting toxicity of high-dose chemotherapy (HDCT), particularly alkylate agent-associated myelosuppression^29^. NTZ can be used to avoid HDCT because of its wide therapeutic window and safety in normal tissues, indicating its promise in clinical applications.

We then found that the tumor suppressor gene ING1 was the target of NTZ through high-throughput screening and might be associated with cell cycle regulation by bioinformatics analysis. In previous studies, downregulation of ING1 promoted glioma growth and progression by accelerating G1 phase transit and resulted in rapid apoptosis of glioma cells in response to cisplatin; overexpression of ING1 significantly suppressed glioma angiogenesis through upregulation of Ang1 and Ang4^30,31^. Consistent with these results, our survival analysis showed that low expression of ING1 predicted a poor prognosis in GBM patients. Subsequently, we confirmed that NTZ reduced glioma cell viability by upregulating ING1-induced cell cycle arrest in glioma, which illustrates the core role of ING1 in NTZ treatment. In addition, ING1 is an epigenetic regulator of H3K4me3 that recruits the mSin3A/HDAC1/2 complex, which possesses crucial deacetylase activity for growth inhibition of p33ING1b^32^. Significant expression of nuclear HDAC1 is associated with recurrence, progression and advanced grade, particularly poor overall survival in glioma^33,34^. Similarly, HDAC1 knockdown inhibits glioma cell proliferation and invasion and induces apoptosis^33,34^. And HDAC1 inhibitors are widely tested as single or combined agents in clinical trials for multiple types of cancer, including GBM^35,36^. RGFP109, a selective inhibitor against HDAC1 and HDAC3, overcomes temozolomide resistance by suppression of NF-κB pathway^37^. The presence of HDAC1 inhibitor, Butyrate, also can enhance irradiation induced cell apoptosis in glioma^38^. However, whether the association between ING1 and HDAC1 exists in glioma, or even after NTZ treatment has not been investigated nowadays. In our study, we found that NTZ inhibited the expression of HDAC1, however, only knockdown of ING1 did not affect HDAC1 level. Interesting, combination of NTZ and si-ING1 increased HDAC1 expression compared with NTZ-treated group. Previous study showed the N terminus of p33ING1b, a divergent member of ING1 polypeptide family, can inhibit cell growth in a manner dependent on the intact Sin3-HDAC-interacting domain^32^. This indicates that ING1 might regulate HDAC1 expression beyond an epigenetic mechanism under the context of NTZ in glioma, which should be confirmed in further studies.

Protein level can be increased by two major mechanisms: enhanced generation and inhibited degradation. Through the high-throughput screening and western blotting analysis, we found that NTZ increased the transcription of ING1, but whether it reduces ING1 degradation remains unclear. Autophagy is a highly conserved homeostatic and lysosomal degradation process in mammalian cells^14,17^. Autophagy begins with the formation of double-membrane autophagosomes, followed by trafficking to lysosomes, and the outer membrane of the autophagosomes fuses with lysosomes to form single-membrane autolysosomes, in which “waste” proteins and cellular organelles are recycled^14^. A previous study reported that NTZ and its active metabolite TZO inhibited the quinone oxidoreductase NQO1 through stimulation of autophagy and suppression of the mTORC1 signaling pathway in *M. tuberculosis* infection^11^. However, it is not completely clear how NTZ-associated autophagy influences the inhibition of glioma growth, even with ING1 degradation. Notably, we found that autophagosomes accumulated in a time-dependent manner as shown by the GFP-RFP-LC3 fluorescence assay. Furthermore, similar to Bafilomycin A1 and chloroquine, NTZ impaired late-staged acidification of autophagosomes in lysosomes with increased expression of SQSTM1 and LC3 II, which suggests a novel role of NTZ as an autophagy inhibitor in glioma. Blockage of late-stage autophagy resulted in ING1 upregulation, which provides an explanation for the reduced ING1 degradation after NTZ treatment.

Importantly, autophagy may act as a “pro-survival” or “pro-death” response to specific biological and experimental contexts in different types of mammalian cells^18,39^. Under moderate stimuli, autophagy is a “pro-survival” signal that maintains cell survival by supplying metabolic substrates^40^. Conversely, excessive or uncontrolled autophagy as a “pro-death” signal contributes to a cell death with or without apoptosis^39,41^. Thus, the contradictory and heterogeneous roles of autophagy depend on unique stresses that should be evaluated in specific diseases^14^. In glioma, chemotherapy and radiation-induced autophagy have become potential therapeutic strategies^16,42^. Chloroquine, an autophagy inhibitor, in combination with temozolomide significantly increased the levels of LC3-II, CHOP/GADD-153, and cleaved PARP, and triggered ER stress, which potentiated temozolomide-induced cytotoxicity^43,44^. Rapamycin, an inhibitor of the mTOR signaling pathway, promoted malignant glioma cell death and sensitized glioma to combined radiotherapy or temozolomide treatment by autophagic activation along with increased expression of Beclin-1, Atg5 and LC3-II^45^. Our results demonstrated that chloroquine synergistically enhanced the chemosensitivity of glioma cells toward NTZ by the inhibition of ING1 autophagic degradation, which suggests a novel therapeutic strategy for clinical treatment of glioma.

As noted previously, the BBB is a major challenge for delivering drugs to glioma cells, and the ability of NTZ to cross the BBB needs to be determined. Although NTZ exhibits a favorable toxicity profile, its relatively high lipid solubility indicates the potential capacity of BBB penetration. Our result showed that NTZ could penetrate BBB towards the glioma in the orthotopic xenograft model and prolong the survival time of nude mice bearing glioma in vivo. And LC-MS data demonstrated there existed TZO in the tumor tissue after NTZ treatment, which also indicates the BBB penetrability of NTZ in vivo. In the other regard, therapeutic nanoparticles can be designed to aid the transport of agents, antibodies, and photosensitizers through morphophysiological barriers and improve penetrating capacity of drugs in different tumors^46,47^. Recent studies have shown that nano-delivery systems enhance the penetrability of NTZ. Among these, laser-responsive NTZ-zinc phthalocyanine-liposome nanoparticles (^125^I-NTZ-ZnPc-LPs) possess selective tumor targeting with a high accumulation of radioactivity in the liver^48^. To date, few biodistribution of NTZ nanoparticles in the brain are investigated in recent studies. Taken together, the higher BBB penetrability and more efficient concentration of NTZ with less side effects prompted us to explore and develop more advanced NTZ nanoparticles for glioma chemotherapy and photodynamic therapy in our next study.

**Fig. 7:**
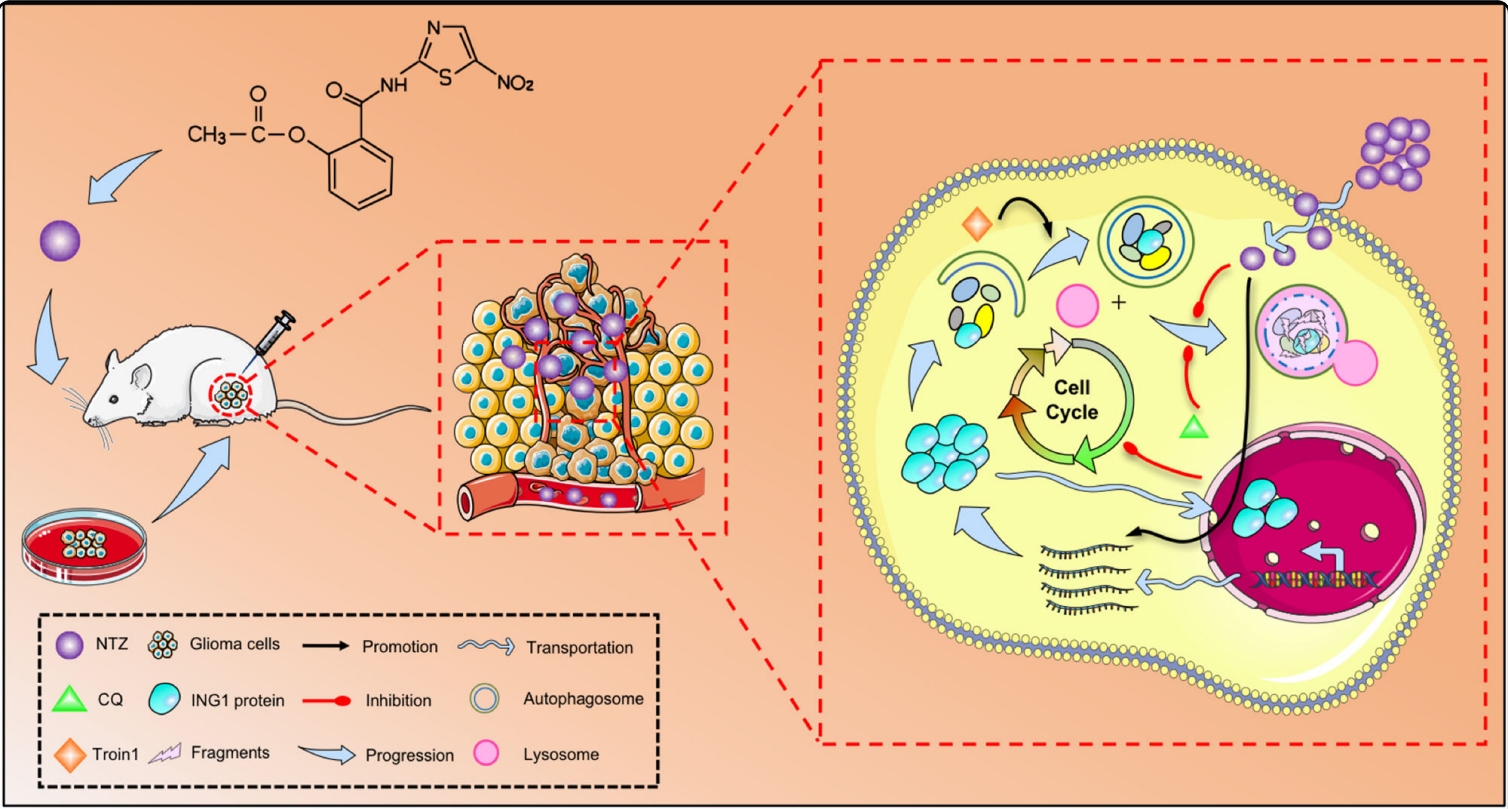
Schematic model for the molecular mechanism of NTZ in glioma chemotherapy

In conclusion, our study reveals a new underlying mechanism of NTZ chemotherapy (Fig. 7). We showed that NTZ effectively induces cell cycle arrest in glioma and inhibits glioma growth in vitro and vivo by blockage of late-stage autophagy with a favorable toxicity profile. ING1, a potential target of NTZ, exerts its antitumor enhancing effect on chloroquine through decreased autophagic degradation. This study provides a novel mechanistic basis and strategy for clinical application of NTZ for glioma in the future.

## Materials and methods

### Cell lines and chemical reagents

Human glioblastoma cell lines (LN229, A172, U87, and HUVEC) were obtained from China Infrastructure of Cell Line Resource (National Science and Technology Infrastructure, NSTI). Chloroquine was purchased from Sigma-Aldrich (St. Louis, MO, USA). Torin 1, Bafilomycin A1, and NTZ were all purchased from MedChem Express (MCE, USA).

### Cell viability and clone formation assay

GBM cells were seeded in 96-well plates with a density of 1 × 10^5^ cells/well and treated with 20 μl MTT as indicated at 48 h or 72 h. GBM cells were seeded in six-well plates with a density of 500 cells/well and treated with NTZ for 2 weeks.

### Transmission electron microscopy (TEM)

The treated cells were fixed with 2.5% glutaraldehyde at 4 °C and post-fixed with 1% osmium tetroxide following by increasing dehydration in ethanol and acetone.

### Cell cycle analysis

LN229 cells at a density of 5 × 10^5^ cells/well were exposed to NTZ and stained with PI/RNase staining buffer at 4 °C for 30 min for flow cytometric analysis.

### Immunofluorescence staining

A total of 1 × 10^5^ LN229 cells were treated with NTZ 48 h, fixed with 4% paraformaldehyde, permeabilized with Triton X-100 and blocked with 10% BSA, then incubated with the Ki67 primary antibody overnight at 4 °C, followed by florescent secondary antibody, as well as DAPI and determined by the fluorescence microscope in the next day.

### Western blotting

Total cell protein was extracted and lysed in RIPA buffer with protease inhibitor. The lysates were separated by 12.5% SDS-PAGE gels, transferred to PVDF membranes, and then blocked in 5% skim milk with TBST for 2 h and incubated with primary antibodies at 4 ℃ overnight. After incubation with secondary antibodies, immunoreactive complexes were visualized using ChemiDoc^TM^ MP System.

### RNA-sequencing (RNA-seq) and TCGA data analysis

Total RNA was extracted using TRIzol reagent following the manufacturer’s procedure. Then, the cleaved RNA fragments were reverse-transcribed to create the final cDNA library in accordance with the protocol for the mRNASeq sample preparation kit. In addition, we performed paired-end sequencing on an Illumina Hiseq X10 system following the vendor’s recommended protocol. The TCGA data used in this study were downloaded from https://cancergenome.nih.gov.

### Small interfering RNA transfection

LN229 cells were transfected with ING1 siRNA (siING1- F, 5′-GCGCAAUAACUGAGAUCCUTT-3′; siING1-R, 5′-AGGAUCUCAGUUAUUGCGCTT-3′) and negative control siRNA (negative control-F, 5′-UUCUCCGAACGUGUCACGUTT-3′; negative control-R, 5′-ACGUGACACGUUCGGAGAATT-3′) using X-tremeGENE siRNA Transfection Reagent according to the manufacturer’s instruction.

### Co-immunoprecipitation (Co-IP)

Cell protein lysates were incubated with 20 μl Protein A/G Plus-Agarose at 4 ℃ for 2 h and then incubated with primary 2 μg antibody and 60 μl Protein A/G Plus-Agarose at 4 ℃ overnight. Beads-Primary Antibody-Protein complex was washed by gentle pipetting using washing buffer. After the supernatant removement, samples were resuspended with 200 μl washing buffer, centrifuged for 8 times and harvested with RIPA and 1× loading buffer for western blotting.

### GFP-RFP-LC3 lentivirus transfection and fluorescence imaging

LN229 cells were transfected with GFP-RFP-LC3 lentivirus for 24 h to detect autophagic flux according to the manufacturer’s protocol. After incubation for 12 h, the cells were treated with NTZ for additional 24 h and 48 h. The fluorescence was determined using the laser confocal microscope by which the autophagosomes (yellow dots) and autolysosomes (red dots) were captured.

### Real-time PCR

Total RNA was purified from LN229 cells using the Trizol reagent and reverse transcribed using TaqMan® MicroRNA Reverse Transcription Kit. Real-time PCR was carried out on a 7900 Fast Realtime System using TaqMan® Gene Expression Master Mix. The ING1 and GAPDH primers were: ING1-F, 5′-TGGAGGAAGCGGAAAGC-3′; ING1-R, 5′-CTTGCTGTTGGGCTTGTC-3′; GAPDH-F, 5′-ACCACAGTCCATGCCATCAC-3′; GAPDH-R, 5′-TCCACCACCCTGTTGCTGTA-3′.

### Immunohistochemistry

Formalin-fixed samples were embedded with paraffin and sliced into 5 μm thick sections. Then sample sections were immunostained for ING1, LC3, and SQSTM1 primary antibodies at 4 °C overnight and secondary antibodies at 37 °C for 30 min. Next, samples were visualized by using the diaminobenzidine (DAB) substrate kit for 10 min. After intensive washing, samples were counterstained with haematoxylin, then dehydrated and coverslipped according to manufacturer’s protocol.

### Liquid chromatography-mass spectrometry (LC-MS)

Chromatographic separation was performed using an X-Terra C18 analytical column. The mobile phase of ammonium acetate aqueous (10 mM, pH 3.5)–acetonitrile (20:80, v/v) was applied at a flow rate of 1.0 ml/min. The 0.3 g brain samples were spiked with 100 μl of nifuroxazide (5 ug/ml) as IS and were homogenated with 1 ml 0.9% NaCl. After the addition of 5 ml of acetonitrile for protein precipitation, samples were vortex-mixed for 2 min and centrifuged at 15,000 r.p.m. for 20 min. Supernatant was obtained and 20 μl was injected into the chromatographic system. Calibration curves for TZO fluid were constructed by least-squares linear regression with correlation coefficients of ≥0.998.

### Tumor transplantation model

All 4-week-old BALB/C nude mice were provided by the animal center (Beijing Vital River Laboratory Animal Technology, China) and randomly divided into the control and NTZ groups (6 mice in each group). Then, 100 μl PBS containing 5 × 10^6^ LN229 cells was subcutaneously injected into the right flank of each mouse. NTZ (150 mg/kg/bid) was administered by gavage for 4 weeks. Tumor volume was assessed and calculated every 3 days by (width)^2^ × (length)/2. Mice were sacrificed on day 27, and tumor weight was determined. In orthotopic brain tumor model, a total of 10 μl LN229 suspension containing 1 × 10^6^ cells were injected into caudate nucleus at a depth of 5 mm in BALB/C nude mice. All animal experiments conformed to the European Parliament Directive (2010/63/EU) and were approved by the Institutional Animal Care and Use Committee at Harbin Medical University (No. HMUIRB-2008-06) and the Institute of Laboratory Animal Science of China (A5655-01).

### Statistical analysis

Data from independent experiments are shown as the means ± standard deviations (SD). Statistical analysis between two groups was performed by Student’s *t-*test (two-tailed) and among multiple groups was conducted by one-way ANOVA with SPSS version 18.0 (IBM Analytics, USA). A *P* value < 0.05 was considered statistically significant (*).

## Electronic supplementary material

Table S1

Table S2

Table S3

Table S4

Table S5

Supplementary materials
