# Supplementary material for: Nitazoxanide, an antiprotozoal drug, inhibits late-stage autophagy and promotes ING1-induced cell cycle arrest in glioblastoma
Source: Cell Death Dis. 2018 Oct 9;9(10):1032. doi: 10.1038/s41419-018-1058-z (PMC6177448; doi:10.1038/s41419-018-1058-z)
Supplement: Supplementary file 5 — Table S5 [file 41419_2018_1058_MOESM5_ESM.docx]

**Table S5.** TZO brain concentration after the administration of 150 mg/kg NTZ.

| Group | TZO brain concentration(ug/kg) |
| --- | --- |
| control-1 | 0 |
| control-2 | 0 |
| control-3 | 0 |
| control-4 | 0 |
| Nitazoxanide-1 | 29.5 |
| Nitazoxanide-2 | 26.4833 |
| Nitazoxanide-3 | 25.008 |
| Nitazoxanide-4 | 23.7592 |

Tizoxanide, TZO.
