# Supplementary material for: Nitazoxanide, an antiprotozoal drug, inhibits late-stage autophagy and promotes ING1-induced cell cycle arrest in glioblastoma
Source: Cell Death Dis. 2018 Oct 9;9(10):1032. doi: 10.1038/s41419-018-1058-z (PMC6177448; doi:10.1038/s41419-018-1058-z)
Supplement: Supplementary file 6 — Supplementary materials [file 41419_2018_1058_MOESM6_ESM.docx]

**Supplementary materials**

**Table S1.** Differential transcript expression by RNA-seq analysis.

**Table S2.** Differential gene expression by RNA-seq analysis.

**Table S3.** GO enrichment of altered genes.

**Table S4.** KEGG enrichment of altered genes.

**Table S5.** TZO brain concentration after the administration of 150 mg/kg NTZ.
